# Composite Dietary Antioxidant Index Is Negatively Associated with Hyperuricemia in US Adults: An Analysis of NHANES 2007–2018

**DOI:** 10.1155/2023/6680229

**Published:** 2023-08-17

**Authors:** Zhenzong Lin, Haokai Chen, Qiwen Lan, Yinghan Chen, Wanzhe Liao, Xuguang Guo

**Affiliations:** ^1^Department of Clinical Laboratory Medicine, Guangdong Provincial Key Laboratory of Major Obstetric Diseases, Guangdong Provincial Clinical Research Center for Obstetrics and Gynecology, The Third Affiliated Hospital of Guangzhou Medical University, Guangzhou 510150, China; ^2^Department of Clinical Medicine, The Third Clinical School of Guangzhou Medical University, Guangzhou, China; ^3^Guangdong Provincial Key Laboratory of Major Obstetric Diseases, The Third Affiliated Hospital of Guangzhou Medical University, Guangzhou, China; ^4^Key Laboratory of Reproduction and Genetics of Guangdong Higher Education Institutes, The Third Affiliated Hospital of Guangzhou Medical University, Guangzhou, China; ^5^Department of Medical Imageology, The Second Clinical School of Guangzhou Medical University, Guangzhou 511436, China; ^6^Department of Clinical Medicine, The Nanshan College of Guangzhou Medical University, Guangzhou 511436, China; ^7^Guangzhou Key Laboratory for Clinical Rapid Diagnosis and Early Warning of Infectious Diseases, KingMed School of Laboratory Medicine, Guangzhou Medical University, Guangzhou, China

## Abstract

Hyperuricemia and its complications are severe risks to human health. Dietary intervention is considered an essential part of the management of hyperuricemia. Studies have reported that the intake of antioxidants has a positive effect on hyperuricemia. Here, we collected data from 8761 participants of the National Health and Nutrition Examination Survey for this analysis. Daily intakes of vitamins A, C, and E; manganese; selenium; and zinc were calculated as the composite dietary antioxidant index (CDAI). The participants were divided into four groups (Q1, Q2, Q3, and Q4) according to the CDAI. Univariate analysis was used to assess the association of covariates with hyperuricemia. The association between the CDAI and hyperuricemia was evaluated using multinomial logistic regression, and its stability was determined by stratified analysis. Our results revealed that the CDAI has a significant negative association with hyperuricemia (Q2: 0.81 (0.69, 0.95); Q3: 0.75 (0.62, 0.90); Q4: 0.65 (0.51, 0.82); *P* < 0.01). The results of stratified analysis emphasize that this association between CDAI and hyperuricemia is stable. In conclusion, this study suggested a negative association between the CDAI and hyperuricemia.

## 1. Introduction

Hyperuricemia (HUA) is clinically defined as high levels of serum uric acid in the body (>7 mg/dL for men and >6 mg/dL for women). Excess uric acid is often deposited in the joint, causing gout [1]. In addition, numerous complications associated with hyperuricemia have been reported, such as chronic kidney disease, cardiovascular disease, type 2 diabetes, and hypertension, and are considered critical burdens on human health [2–5]. Research data have shown that the prevalence of HUA among adults in the US is 20.2% for men and 20% for women [6]. Even worse, there is no curative treatment available [7]. In general, dietary intervention is considered to play a critical role in the management of HUA.

Recent studies have shown that excess uric acid in HUA patients can locally activate oxidative stress [8]. Oxidative stress produces oxidants, and the accumulation of oxidants leads to DNA oxidation, causing abnormal apoptosis and organ dysfunction and consequently leading to the abovementioned complications [8, 9]. Thus, there may be an interaction mechanism between uric acid and antioxidants, and antioxidants may mitigate the damage caused by uric acid.

Vitamin (vit) C, a common antioxidant, has been shown to have a negative association with HUA. Sun et al. also reported that supplementation with vit C might delay the development of hyperuricemic nephropathy [10, 11]. In addition, vit A, vit E, and zinc (Zn) have been shown to reduce the level of serum uric acid [12–16]. Although numerous studies have reported the effect of a single antioxidant on HUA, a study evaluating the association between comprehensive antioxidants and HUA still needs to be completed.

According to previous studies, we proposed a hypothesis that the intake of antioxidants and the risk of hyperacidity are inversely associated. We constructed a composite dietary antioxidant index (CDAI) consisting of food parameters for vit A, C, and E; manganese (Mn); selenium (Se); and Zn to represent an individual's antioxidant intake status and to enhance the credibility of disease risk assessment [17]. A cross-sectional study including 8761 participants was conducted based on the National Health and Nutrition Examination Survey (NHANES) from 2007 to 2018 to investigate the association between the CDAI and HUA.

## 2. Methods and Materials

### 2.1. Data Sources and Study Design

Subject data for this study were obtained from the NHANES 2007–2018. NHANES is a representative cross-sectional survey of the American population designed to collect nutrition and health information from noninstitutionalized people. The NHANES website provides comprehensive and detailed information on study design, demographics, dietary assessments, health interviews, physical examinations, and laboratory data. Demographic and health-related information was obtained through questionnaires. Health interviews were conducted in the participants' homes. Dietary assessments were obtained through 24-hour dietary memories. The Mobile Examination Centre was responsible for the physical examination and the collection of blood samples, which were then sent to the laboratory for testing.

**Figure 1:**
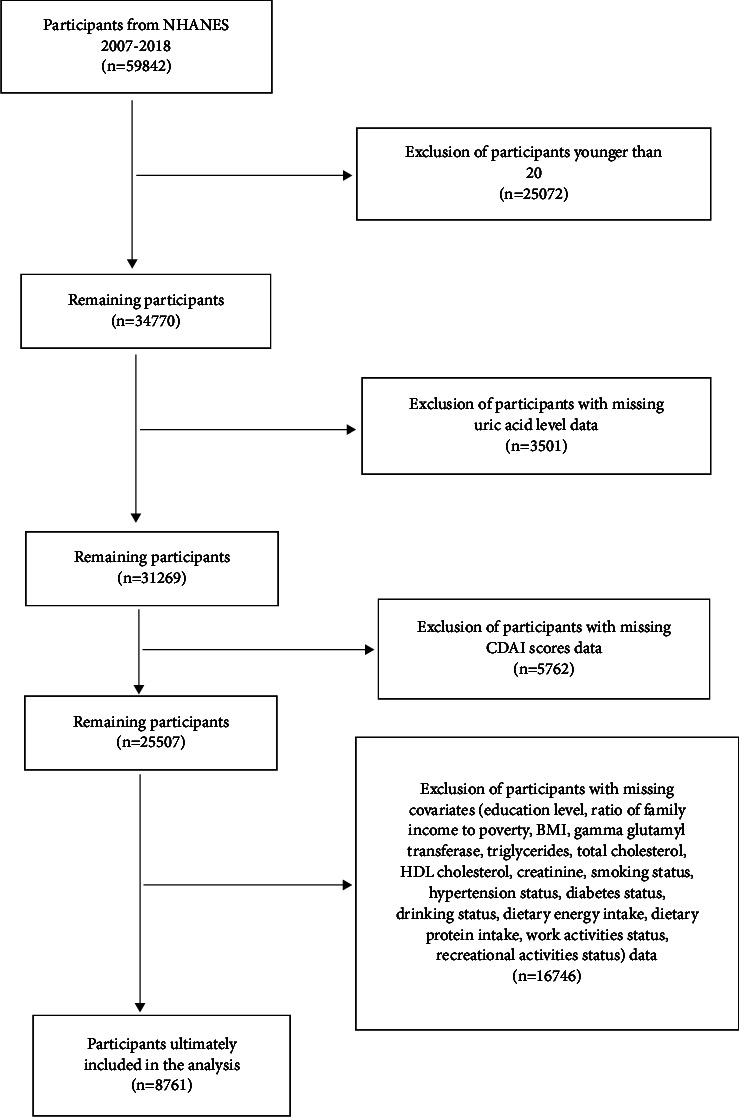
Flowchart for inclusion and exclusion of participants. A total of 59,842 participants in the NHANES during 2007–2018 were included, excluding those younger than 20, those with missing uric acid level data, those with missing CDAI score data, and those with missing covariate data; 8761 people were finally included in the study.

Data marked as missing, refused, and did not know were considered missing data and manually excluded by the researcher. Only participants over 20 years of age were considered for inclusion in the study. After excluding missing data for uric acid levels, CDAI scores and covariates, a total of 8761 participants were included in the final study. The flow graph for inclusion and exclusion is shown in Figure 1. All participants provided written informed consent, and the NCHS Research Ethics Review Committee approved the study (https://wwwn.cdc.gov/nchs/nhanes/default.aspx).

### 2.2. Hyperuricemia and CDAI

HUA was diagnosed as uric acid levels ≥420 *μ*mol/l (7 mg/dL) in males and ≥360 *μ*mol/l (6 mg/dL) in females.

The CDAI proposed by Wright et al. was obtained by a composite calculation of the intake of multiple dietary antioxidants [17–20]. For all participants included in this study, the CDAI contained six dietary antioxidants: vit A, C, and E; Mn; Se; and Zn. The formula for CDAI is **CDAI**=∑_**I**=1_^6^**X**_**i**_ − **μ**_**i**_/**S**_**i**_ [21]. In the formula, *X*_*i*_ is the daily intake of antioxidants, *μ*_*i*_ is the mean of antioxidants in the study population, and *S*_*i*_ is the standard deviation of *μ*_*i*_. In brief, the CDAI is a scoring algorithm based on 24-hour dietary recall data designed to assess the level of antioxidant intake of participants. We averaged participants into four groups based on CDAI, Q1 (−7.177 to −1.178), Q2 (−1.177 to −0.796), Q3 (0.799 to 3.202), and Q4 (3.203 to 88.502).

### 2.3. Covariates

To exclude other factors interfering with the results, age, sex, race, education, household income to poverty ratio, BMI, dietary capacity and protein intake, hypertension status, diabetes status, smoking status, alcohol consumption status, physical activity, and biochemical indicators, including gamma glutamyl transferase, triglycerides, total cholesterol, HDL cholesterol, and creatinine, were selected as covariates for the analysis. Race was categorized as Mexican American, Other Hispanic, Non-Hispanic White, Non-Hispanic Black, and Other race—including multiracial. Educational attainment included three levels: below high school, high school, and above high school. Household income and poverty rates were used to measure participants' household economic status and were categorized into three levels: less than 1, between 1 and 3, and more than 3. Hypertension was defined as participants taking medication for hypertension or having a past/current diagnosis of hypertension. Diabetes was classified into four categories: no, impaired fasting glucose (IFG), impaired glucose tolerance (IGT), and yes. Smoking status was categorized as never, former, and now. Participants who consumed at least 12 alcoholic beverages in a year were considered to have drinking behavior. Physical activity included two categories, work activity status and recreational activity status, with four ratings of no, vigorous, moderate, and both. In addition, specific data for biochemical indicators were provided by the NHANES laboratory. All covariate data for this study can be viewed in detail on the NHANES website (https://www.cdc.gov/nchs/nhanes/index.htm).

### 2.4. Statistical Analyses

The statistical packages R (The R Foundation; https://www.r-project.org; version 3.6.3) and Empower Stats (https://www.empowerstats.net, X&Y solutions, Inc., Boston, Massachusetts) were used to process the data. In the analysis of participant characteristics, continuous variables were expressed as the “mean ± standard deviation,” and categorical variables were expressed as weighted percentages (%). *χ*^2^ tests and Kruskal‒Wallis tests were used to assess the significance of categorical and continuous variables, respectively. Univariate analysis of variance was used to assess the relation between each covariate and HUA. Multinomial logistic regression analysis with five adjusted models was used to investigate the association between CDAI and HUA, and the stability of the association was assessed by stratified analysis. The 95% confidence intervals were calculated. *P* < 0.05 was considered statistically significant in this study.

## 3. Result

### 3.1. Baseline Characteristics of Participants

A total of 8761 participants were included in the analysis (1904 with HUA vs. 6857 with non-HUA). Participants' CDAI ranged from −7.177 to 88.502. Patients with HUA had lower CDAI scores (0.98 ± 3.47 vs. 1.45 ± 3.88, *P* < 0.001). In addition, patients with HUA are more likely to be older, to have a high BMI, and to have hypertension and glucose-related disorders.  Table 1 describes the participants' characteristics in detail.

### 3.2. Analysis of Factors Associated with HUA

In the univariate analysis, several covariates were selected as independent exposure variables in this study to determine the factors that interfered with the association between CDAI and HUA. The results of the univariate analysis indicated that age, sex, race, household income to poverty ratio, marital status, BMI, gamma glutamyl transferase, triglycerides, total cholesterol, HDL cholesterol, creatinine, dietary energy intake, hypertensive status, diabetes status, smoking status, and leisure activity status were statistically significant (*P* < 0.05), demonstrating that they may be potential confounding factors. The results of the univariate analysis are described in detail in Table 2.

### 3.3. Association between CDAI and HUA

**Figure 2:**
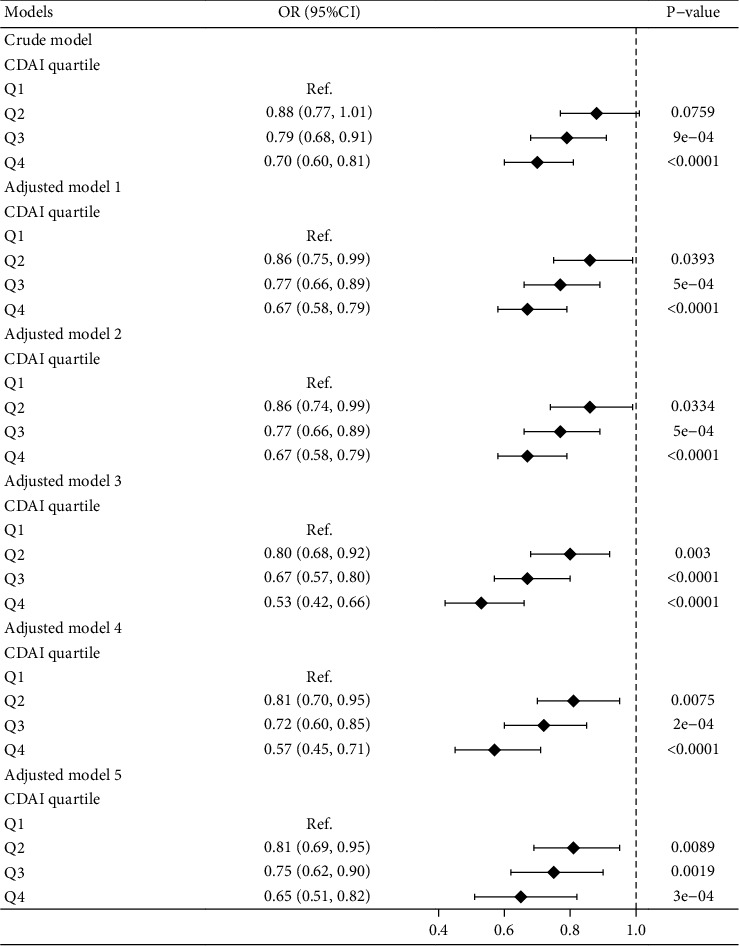
CDAI and HUA have a negative correlation across the different models. A negative association was revealed via a forest map following multinomial logistic regression. *P* < 0.05 was considered significant. The crude model was not adjusted; adjusted model 1 adjusted for age, sex, race, education level, ratio of family income to poverty, and marital status; adjusted model 2 adjusted for model 1 + smoking status, drinking status, work activities status, and recreational activities status; adjusted model 3 adjusted for model 2 + dietary energy intake and dietary protein intake; adjusted model 4 adjusted for model 3 + hypertension status and diabetes status; and adjusted model 5 adjusted for model 4 + BMI, gamma glutamyl transferase, triglycerides, total cholesterol, HDL cholesterol, and creatinine.

**Figure 3:**
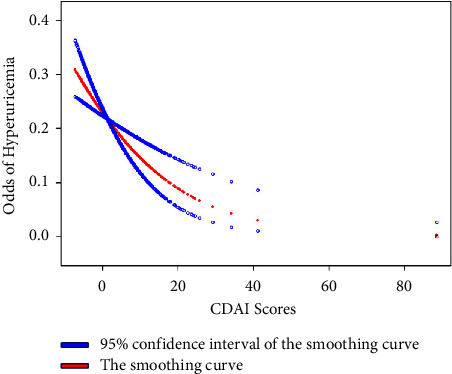
A negative association between CDAI and HUA in adjusted model 5. The negative association between CDAI and HUA in adjusted model 5, which was considered the most accurate model, was visualized as a smoothing curve.

Figure 2 describes the association between the CDAI and HUA. Participants in the study were equally allocated into four groups according to the CDAI. The multinomial logistic regression included one crude and five adjusted models. A negative and statistically significant association of CDAI with HUA was observed in all six models. In model 5, which adjusted for all confounders, participants in the CDAI Q4 group (highest) had a 35% lower risk of suffering from HUA than those in the Q1 group (lowest) (OR = 0.65, 95% CI 0.51, 0.82, *P* < 0.05). To visualize the association between the CDAI and HUA, smoothed curve fits were plotted according to adjusted model 5. The results are shown in Figure 3, where the CDAI is negatively associated with HUA.

### 3.4. Stratification Analysis

Statistically significant covariates from the univariate analysis were included in the stratified analysis to assess the stability of the association between CDAI and HUA in different populations. All covariates in the stratified analysis except for the stratified variables were adjusted. CDAI shows a negative association with HUA in the vast majority of subgroups, except in the few subgroups where a positive association is observed. In addition, no statistically significant results are observed for any subgroups with positive associations. The results suggest that the CDAI has stability in its negative association with HUA and may be a valid protective factor for HUA.  Table 3 describes the detailed results of the stratified analysis.

## 4. Discussion

This research analysed the most representative US population data (4244 males and 4571 females) and found a negative association between CDAI and the incidence of HUA in the population, which confirmed our hypothesis. These results suggest that appropriate modifications in the level and proportion of antioxidants in the diet may facilitate the prevention and treatment of HUA.

This is the first large-scale study to consider the association between a composite of dietary antioxidants and HUA. Although previous studies have researched the effects of dietary antioxidants such as vit C, vit E, and Zn on HUA, they have not been considered in combination. In the average person's daily diet, it is clear that a single antioxidant intake is difficult to achieve, and it is more likely that a variety of foods and multiple antioxidants are absorbed. In addition, there is no single antioxidant component in food, for example, tomatoes, are rich in vit A, vit E, vit C, Mn, and many other antioxidants [22, 23]. Therefore, a comprehensive study on the effects of dietary antioxidants on HUA is necessary.

The composition of dietary antioxidants includes vit A, C, and E; Mn; Se; and Zn. There is a large amount of research in the field reporting the association, causality, and mechanism of the effect of individual nutrients on HUA. A study of 1387 males showed a negative association between vit C intake and HUA [24]. Studies by Huang et al. also showed that vit C intake can reduce serum uric acid levels and prevent the development of gout [25, 26]. Current reports in the field of vit C reducing serum uric acid focus on the renal excretion mechanism. Two vit C transport proteins, SLC23A1 and SLC23A2, existing in proximal renal tubular epithelial cells, can alter the activity of URAT1 in renal tubular cells, thus promoting uric acid excretion and reducing serum uric acid levels [27–31]. The mechanism of xanthine oxidase (XO) inhibition is also of concern. An *in vitro* study reported that vit C and vit E could inhibit XO activity. Studies have also suggested a negative association between vit E and HUA [12, 13, 32]. Similar associations and mechanisms have also been reported for dietary Zn [14, 33–35]. The role of Se in HUA is currently controversial, with some studies claiming a negative association between serum selenium and uric acid levels [15, 36]. However, other studies have suggested the opposite. The difference may be related to the source of Se [37]. This suggests that we consider the role of other substances in food while increasing the dietary intake of antioxidants. Ma et al. showed a negative association between Mn and uric acid levels [16].

There is a near consensus that XO plays a crucial role in uric acid metabolism. Uric acid is generated during the XO-catalyzed conversion of hypoxanthine and xanthine and is accompanied by the production of reactive oxygen species (ROS) [38]. Furthermore, uric acid increases the production of inflammatory factors and reduces the amount of free radical nitric oxide (NO) in cells, which can lead to an increase in ROS levels and oxidative stress, with the contribution of XO [39, 40]. This process can activate osteoclasts and inhibit osteoblasts, resulting in increased bone loss and triggering osteoporosis and bone destruction [39, 41–43]. Several antioxidants have been reported to function as XO inhibitors, reducing serum uric acid levels and scavenging intracellular oxygen radicals, thereby ameliorating the impairment of hyperuricemia. Zeng et al. showed that baicalein and baicalein, two antioxidants widely present in plants, could bind to the FAD center of XO and inhibit XO activity, suppressing uric acid production and oxidative stress levels [38]. Ellagic acid, another natural antioxidant, has also been found to inhibit XO and scavenge superoxide anions (O^2−^), ameliorating hyperuricemia [44]. Moreover, the inhibition of XO also contributes to increased bone formation by promoting osteoblast differentiation, which may be associated with the inhibition of ROS [39, 45–47]. We hypothesize that the association of a high CDAI with a low risk of hyperuricemia may be attributable to the inhibition of dietary antioxidants on XO. In fact, Li et al. showed that vit C supplementation reduced XO activity in hyperuricemic rats and decreased ROS levels by inhibiting TGF-beta [11]. Similar findings have also been reported in other studies [12, 44, 48, 49]. However, studies exploring the inhibitory capacity of these antioxidants against XO and their mechanisms are still insufficient. Our findings may provide valuable insights for further research.

In this study, the association between the CDAI and hyperuricemia was explored through a cross-sectional study that included 8761 participants. Different models were constructed to exclude the effect of confounding factors on the results. The results showed that the negative association between CDAI and hyperuricemia was stable across the different models. In the final adjusted model 5, the Q4 group (highest) had a 35% lower risk of suffering from HUA than the Q1 group (lowest) (OR = 0.65, 95% CI 0.51, 0.82, *P* < 0.05). This suggests that the treatment of hyperuricemia may be facilitated by adjusting the ratio of antioxidants in the diet.

To summarize, the CDAI is negatively associated with HUA. However, there are still some limitations to our study. First, we cannot determine the causal relation between the CDAI and HUA, which is limited because our research is a cross-sectional study. Although a large number of studies have shown that dietary antioxidants reduce serum uric acid levels, further cohort studies and clinical trials are needed to determine the causal relationship between CDAI and HUA and the therapeutic value of CDAI. Second, although a large number of participants were included in this study, it was limited to US residents only. Considering the differences between a variety of factors, such as body composition, lifestyle, and dietary habits of residents in different countries and regions, multicenter controlled trials are needed to validate our findings.

## 5. Conclusion

Our study suggests that there is a significant and stable negative association between CDAI and HUA in the population. This suggests that the CDAI may be an effective protective factor for HUA and holds promise as a preventive and therapeutic tool for HUA.

## Figures and Tables

**Table 1:** Characteristics of study participants from NHANES 2007–2018.

Characteristics	Hyperuricemia		*P* value
	No	Yes	
*N*	6857	1904	
Age (year), mean ± SD	49.13 ± 17.39	55.11 ± 17.30	<0.001
Sex			<0.001
Male	3253 (47.44%)	991 (52.05%)	
Female	3604 (52.56%)	913 (47.95%)	
Race			<0.001
Mexican American	1053 (15.36%)	195 (10.24%)	
Other Hispanic	728 (10.62%)	142 (7.46%)	
Non-Hispanic White	3210 (46.81%)	978 (51.37%)	
Non-Hispanic Black	1237 (18.04%)	413 (21.69%)	
Other race—including multiracial	629 (9.17%)	176 (9.24%)	
Education level			0.181
<High school	1579 (23.03%)	418 (21.95%)	
High school	1524 (22.23%)	460 (24.16%)	
>High school	3754 (54.75%)	1026 (53.89%)	
Ratio of family income to poverty			0.062
≤1	1442 (21.03%)	367 (19.28%)	
1–3	2830 (41.27%)	840 (44.12%)	
>3	2585 (37.70%)	697 (36.61%)	
Marital status			<0.001
Married/cohabiting	4411 (64.33%)	1195 (62.76%)	
Widowed/divorced/separated	1451 (21.16%)	495 (26.00%)	
Never married	995 (14.51%)	214 (11.24%)	
BMI (kg/m^2^)			<0.001
<20	355 (5.18%)	19 (1.00%)	
20–25	1881 (27.43%)	237 (12.45%)	
25–30	2370 (34.56%)	553 (29.04%)	
≥30	2251 (32.83%)	1095 (57.51%)	
Gamma glutamyl transferase (IU/L), mean ± SD	26.77 ± 33.05	37.87 ± 59.33	<0.001
Triglycerides (mg/dL), mean ± SD	118.54 ± 100.09	155.26 ± 131.07	<0.001
Total cholesterol (mg/dL), mean ± SD	191.54 ± 41.25	197.05 ± 43.15	<0.001
HDL cholesterol (mg/dL), mean ± SD	54.86 ± 16.15	50.03 ± 15.23	<0.001
Creatinine (mg/dL), mean ± SD	0.86 ± 0.48	1.03 ± 0.56	<0.001
Dietary energy intake (kcal)	4081.69 ± 1617.59	3899.22 ± 1569.76	<0.001
Dietary protein intake (gm)	160.98 ± 69.39	157.66 ± 68.57	0.064
CDAI	1.45 ± 3.88	0.98 ± 3.47	<0.001
Hypertension status			<0.001
No	4200 (61.25%)	713 (37.45%)	
Yes	2657 (38.75%)	1191 (62.55%)	
Diabetes status			<0.001
No	4416 (64.40%)	832 (43.70%)	
Yes	1284 (18.73%)	625 (32.83%)	
IFG	535 (7.80%)	247 (12.97%)	
IGT	622 (9.07%)	200 (10.50%)	
Smoking status			<0.001
Never	3819 (55.69%)	1004 (52.73%)	
Now	1377 (20.08%)	314 (16.49%)	
Former	1661 (24.22%)	586 (30.78%)	
Work activities status			0.752
No	3989 (58.17%)	1131 (59.40%)	
Vigorous	266 (3.88%)	67 (3.52%)	
Moderate	1558 (22.72%)	421 (22.11%)	
Both	1044 (15.23%)	285 (14.97%)	
Recreational activities status			<0.001
No	3409 (49.72%)	1093 (57.41%)	
Vigorous	554 (8.08%)	107 (5.62%)	
Moderate	1890 (27.56%)	513 (26.94%)	
Both	1004 (14.64%)	191 (10.03%)	
Drinking status			0.765
Yes	4972 (72.51%)	1374 (72.16%)	
No	1885 (27.49%)	530 (27.84%)	

CDAI: composite dietary antioxidant index; BMI: body mass index; IFG: impaired fasting glycemia; IGT: impaired glucose tolerance. *P* < 0.05 was considered statistically significant.

**Table 2:** Univariate analysis of the factors associated with hyperuricemia in participants from NHANES 2007–2018.

Exposure variables	Hyperuricemia	
	OR (95% CI)	*P* value
Age (years)	1.02 (1.02, 1.02)	<0.0001
Sex		
Male	Ref.	
Female	0.83 (0.75, 0.92)	0.0004
Race		
Mexican American	Ref.	
Other Hispanic	1.05 (0.83, 1.33)	0.6662
Non-Hispanic White	1.65 (1.39, 1.95)	<0.0001
Non-Hispanic Black	1.80 (1.49, 2.18)	<0.0001
Other race—including multiracial	1.51 (1.20, 1.89)	0.0004
Education level		
<High school	Ref.	
High school	1.14 (0.98, 1.32)	0.0864
>High school	1.03 (0.91, 1.17)	0.6252
Ratio of family income to poverty		
≤1	Ref.	
1–3	1.17 (1.02, 1.34)	0.029
>3	1.06 (0.92, 1.22)	0.4252
Marital status		
Married/cohabiting	Ref.	
Widowed/divorced/separated	1.26 (1.12, 1.42)	0.0002
Never married	0.79 (0.68, 0.93)	0.0049
BMI (kg/m^2^)		
<20	Ref.	
20–25	2.35 (1.46, 3.81)	0.0005
25–30	4.36 (2.72, 6.98)	<0.0001
≥30	9.09 (5.70, 14.50)	<0.0001
Gamma glutamyl transferase (IU/L)	1.01 (1.00, 1.01)	<0.0001
Triglycerides (mg/dL)	1.00 (1.00, 1.00)	<0.0001
Total cholesterol (mg/dL)	1.00 (1.00, 1.00)	<0.0001
HDL cholesterol (mg/dL)	0.98 (0.98, 0.98)	<0.0001
Creatinine (mg/dL)	2.51 (2.12, 2.98)	<0.0001
Dietary energy intake (kcal)	1.00 (1.00, 1.00)	<0.0001
Dietary protein intake (gm)	1.00 (1.00, 1.00)	0.0638
CDAI	0.96 (0.95, 0.98)	<0.0001
Hypertension status		
No	Ref.	
Yes	2.64 (2.38, 2.93)	<0.0001
Diabetes status		
No	Ref.	
Yes	2.58 (2.29, 2.92)	<0.0001
IFG	2.45 (2.07, 2.90)	<0.0001
IGT	1.71 (1.43, 2.03)	<0.0001
Smoking status		
Never	Ref.	
Now	0.87 (0.75, 1.00)	0.0478
Former	1.34 (1.19, 1.51)	<0.0001
Recreational activities status		
No	Ref.	
Vigorous	0.60 (0.48, 0.75)	<0.0001
Moderate	0.85 (0.75, 0.95)	0.0061
Both	0.59 (0.50, 0.70)	<0.0001
Work activities status		
No	Ref.	
Vigorous	0.89 (0.67, 1.17)	0.4005
Moderate	0.95 (0.84, 1.08)	0.4555
Both	0.96 (0.83, 1.11)	0.6127
Drinking status		
Yes	Ref.	
No	1.02 (0.91, 1.14)	0.765

CDAI: composite dietary antioxidant index; BMI: body mass index; IFG: impaired fasting glycemia; IGT: impaired glucose tolerance. *P* < 0.05 was considered statistically significant.

**Table 3:** Stratified analysis of the association between CDAI and hyperuricemia in adjusted model 5.

Stratified variables	*N*	CDAI quartile						
		Q1	Q2 (OR 95% CI)	*P* value	Q3 (OR 95% CI)	*P* value	Q4 (OR 95% CI)	*P* value
Age								
20–40	2884	Ref.	1.01 (0.72, 1.42)	0.9523	0.76 (0.52, 1.10)	0.1431	0.79 (0.50, 1.26)	0.3187
41–59	2805	Ref.	0.91 (0.67, 1.24)	0.5603	0.90 (0.64, 1.27)	0.5507	0.82 (0.53, 1.26)	0.3613
60–80	3072	Ref.	0.74 (0.58, 0.94)	0.0128	0.74 (0.56, 0.99)	0.0391	0.53 (0.36, 0.78)	0.0011
Sex								
Male	4244	Ref.	0.70 (0.55, 0.90)	0.0057	0.71 (0.54, 0.92)	0.0092	0.58 (0.42, 0.80)	0.0008
Female	4517	Ref.	0.92 (0.74, 1.15)	0.4615	0.78 (0.59, 1.03)	0.0857	0.75 (0.52, 1.10)	0.1382
Race								
Mexican American	1248	Ref.	0.98 (0.60, 1.62)	0.9397	0.98 (0.56, 1.71)	0.9523	0.88 (0.43, 1.81)	0.7277
Other Hispanic	870	Ref.	0.33 (0.17, 0.63)	0.0008	0.38 (0.18, 0.79)	0.0096	0.36 (0.14, 0.93)	0.0346
Non-Hispanic White	4188	Ref.	0.85 (0.67, 1.07)	0.169	0.87 (0.67, 1.13)	0.2878	0.72 (0.52, 1.01)	0.0577
Non-Hispanic Black	1650	Ref.	0.79 (0.57, 1.09)	0.1549	0.54 (0.36, 0.81)	0.0028	0.37 (0.22, 0.65)	0.0004
Other race—including multiracial	805	Ref.	1.19 (0.65, 2.20)	0.5704	1.18 (0.62, 2.25)	0.6079	1.52 (0.69, 3.37)	0.2992
Ratio of family income to poverty								
≤1	1809	Ref.	0.72 (0.51, 1.02)	0.0629	0.93 (0.62, 1.38)	0.7094	0.63 (0.36, 1.10)	0.1057
1–3	3670	Ref.	0.80 (0.63, 1.02)	0.0719	0.69 (0.52, 0.91)	0.0088	0.59 (0.41, 0.86)	0.0057
>3	3282	Ref.	0.85 (0.64, 1.14)	0.2866	0.72 (0.53, 0.99)	0.041	0.68 (0.47, 1.01)	0.0544
Marital status								
Married/cohabiting	5606	Ref.	0.89 (0.73, 1.10)	0.2956	0.81 (0.64, 1.02)	0.0761	0.67 (0.50, 0.91)	0.0108
Widowed/divorced/separated	1946	Ref.	0.65 (0.48, 0.88)	0.0054	0.57 (0.40, 0.82)	0.0024	0.54 (0.34, 0.87)	0.0107
Never married	1209	Ref.	0.82 (0.52, 1.31)	0.4155	0.80 (0.47, 1.37)	0.4169	0.80 (0.40, 1.59)	0.5263
BMI (kg/m^2^)								
<20	374	Ref.	0.46 (0.06, 3.50)	0.4563	1.26 (0.16, 9.64)	0.8253	4.81 (0.53, 43.74)	0.1634
20–25	2118	Ref.	0.94 (0.61, 1.44)	0.7762	0.62 (0.38, 1.02)	0.0596	0.65 (0.35, 1.19)	0.1599
25–30	2923	Ref.	0.69 (0.52, 0.92)	0.0116	0.62 (0.45, 0.86)	0.0043	0.35 (0.22, 0.55)	<0.0001
≥30	3346	Ref.	0.84 (0.68, 1.05)	0.1327	0.83 (0.64, 1.06)	0.1352	0.86 (0.62, 1.19)	0.353
Gamma glutamyl transferase (IU/L)								
4–15	2716	Ref.	0.74 (0.52, 1.05)	0.0932	0.73 (0.49, 1.08)	0.1187	0.58 (0.34, 0.97)	0.0378
16–24	2948	Ref.	0.66 (0.50, 0.87)	0.0034	0.65 (0.48, 0.90)	0.0083	0.57 (0.38, 0.86)	0.0071
25–1116	3097	Ref.	0.96 (0.75, 1.22)	0.7258	0.87 (0.66, 1.15)	0.3303	0.75 (0.53, 1.08)	0.1205
Triglycerides (mg/dL)								
14–81	2912	Ref.	0.80 (0.57, 1.13)	0.2123	0.86 (0.59, 1.26)	0.4366	0.73 (0.45, 1.19)	0.2061
82–131	2923	Ref.	0.70 (0.53, 0.92)	0.0118	0.58 (0.42, 0.80)	0.0008	0.57 (0.38, 0.86)	0.0078
132–4233	2926	Ref.	0.91 (0.71, 1.16)	0.4257	0.85 (0.64, 1.12)	0.2358	0.65 (0.45, 0.94)	0.0222
Total cholesterol (mg/dL)								
80–172	2905	Ref.	0.97 (0.72, 1.30)	0.8374	0.90 (0.64, 1.25)	0.518	0.80 (0.52, 1.24)	0.3278
173–206	2889	Ref.	0.73 (0.55, 0.98)	0.0384	0.59 (0.42, 0.83)	0.0023	0.69 (0.45, 1.04)	0.0782
207–612	2967	Ref.	0.76 (0.59, 0.99)	0.0386	0.80 (0.60, 1.08)	0.143	0.53 (0.36, 0.78)	0.0012
HDL cholesterol (mg/dL)								
6–44	2718	Ref.	0.81 (0.61, 1.06)	0.1252	0.97 (0.72, 1.30)	0.8404	0.81 (0.56, 1.18)	0.2784
45–57	2970	Ref.	0.90 (0.68, 1.19)	0.4751	0.76 (0.55, 1.06)	0.1032	0.68 (0.45, 1.03)	0.0705
58–226	3073	Ref.	0.75 (0.56, 1.01)	0.0543	0.54 (0.38, 0.77)	0.0006	0.46 (0.29, 0.74)	0.0013
Creatinine (mg/dL)								
0.32–0.74	2787	Ref.	0.75 (0.53, 1.08)	0.1216	0.81 (0.53, 1.22)	0.3086	0.65 (0.36, 1.14)	0.1322
0.75–0.92	2941	Ref.	1.03 (0.77, 1.37)	0.8503	1.02 (0.73, 1.42)	0.9208	1.01 (0.66, 1.55)	0.9705
0.93–17.41	3033	Ref.	0.75 (0.58, 0.96)	0.0205	0.65 (0.49, 0.85)	0.0021	0.55 (0.39, 0.77)	0.0006
Dietary energy intake (kcal)								
193–3214	2918	Ref.	0.77 (0.61, 0.98)	0.0324	0.83 (0.59, 1.16)	0.272	1.17 (0.68, 2.04)	0.5694
3215–4452	2920	Ref.	0.84 (0.62, 1.15)	0.2758	0.69 (0.49, 0.96)	0.03	0.55 (0.36, 0.83)	0.0049
4453–18959	2923	Ref.	0.56 (0.31, 1.01)	0.0526	0.51 (0.29, 0.90)	0.0193	0.44 (0.24, 0.79)	0.0059
Hypertension status								
No	4913	Ref.	0.90 (0.69, 1.17)	0.4316	0.94 (0.71, 1.26)	0.7	0.80 (0.56, 1.16)	0.2463
Yes	3848	Ref.	0.77 (0.63, 0.94)	0.0112	0.65 (0.51, 0.83)	0.0005	0.59 (0.43, 0.81)	0.001
Diabetes status								
No	5248	Ref.	0.82 (0.65, 1.04)	0.0953	0.62 (0.48, 0.81)	0.0005	0.56 (0.40, 0.79)	0.0007
Yes	1909	Ref.	1.00 (0.75, 1.34)	0.9855	1.06 (0.75, 1.50)	0.7294	0.96 (0.60, 1.52)	0.856
IFG	782	Ref.	0.53 (0.32, 0.90)	0.0178	0.58 (0.33, 1.01)	0.0561	0.47 (0.22, 1.00)	0.0509
IGT	822	Ref.	0.72 (0.42, 1.21)	0.2149	1.07 (0.60, 1.92)	0.821	0.79 (0.37, 1.68)	0.5422
Smoking status								
Never	4823	Ref.	0.76 (0.61, 0.95)	0.0176	0.65 (0.51, 0.84)	0.001	0.60 (0.44, 0.83)	0.0022
Now	1691	Ref.	1.21 (0.83, 1.76)	0.3205	1.32 (0.84, 2.06)	0.2298	1.09 (0.59, 2.01)	0.7916
Former	2247	Ref.	0.72 (0.53, 0.97)	0.0321	0.70 (0.50, 0.98)	0.0388	0.58 (0.38, 0.88)	0.0109
Recreational activities status								
No	4502	Ref.	0.79 (0.64, 0.97)	0.0242	0.79 (0.62, 1.01)	0.0588	0.78 (0.56, 1.08)	0.132
Vigorous	661	Ref.	1.03 (0.45, 2.35)	0.9377	0.35 (0.14, 0.88)	0.0256	0.55 (0.18, 1.68)	0.2931
Moderate	2403	Ref.	0.79 (0.57, 1.09)	0.1551	0.84 (0.59, 1.20)	0.3435	0.59 (0.37, 0.93)	0.0226
Both	1195	Ref.	1.01 (0.57, 1.80)	0.9683	0.64 (0.35, 1.18)	0.1524	0.40 (0.19, 0.81)	0.0114

CDAI: composite dietary antioxidant index; BMI: body mass index; IFG: impaired fasting glycemia; IGT: impaired glucose tolerance. *P* < 0.05 was considered significant.

## Data Availability

The datasets generated and/or analysed during the current study are available in the NHANES repository (https://wwwn.cdc.gov/nchs/nhanes/continuousnhanes/default.aspx?BeginYear=2007, https://wwwn.cdc.gov/nchs/nhanes/continuousnhanes/default.aspx?BeginYear=2009, https://wwwn.cdc.gov/nchs/nhanes/continuousnhanes/default.aspx?BeginYear=2011, https://wwwn.cdc.gov/nchs/nhanes/continuousnhanes/default.aspx?BeginYear=2013, https://wwwn.cdc.gov/nchs/nhanes/continuousnhanes/default.aspx?BeginYear=2015, and https://wwwn.cdc.gov/nchs/nhanes/continuousnhanes/default.aspx?BeginYear=2017).

## Abbreviations

BMI: Body mass index
CDAI: Composite dietary antioxidant index
HUA: Hyperuricemia
IFG: Impaired fasting glucose
IGT: Impaired glucose tolerance
NHANES: National Health and Nutrition Examination Survey
Mn: Manganese
Se: Selenium
Vit: Vitamin
Zn: Zinc.
